# Neoadjuvant chemotherapy and radiotherapy followed by resection/ablation in stage IV rectal cancer patients with potentially resectable metastases

**DOI:** 10.1186/s12885-021-09089-5

**Published:** 2021-12-14

**Authors:** Rongzhen Li, Qiaoxuan Wang, Bin Zhang, Yan Yuan, Weihao Xie, Xiaoxue Huang, Chengjing Zhou, Shu Zhang, Shaoqing Niu, Hui Chang, Dongni Chen, Huikai Miao, Zhi Fan Zeng, Weiwei Xiao, Yuanhong Gao

**Affiliations:** 1 Department of Radiation Oncology, Sun Yat-Sen University Cancer Center, Guangzhou, 510060 People’s Republic of China; 2 State Key Laboratory of Oncology in South China, Collaborative Innovation Center of Cancer Medicine, Guangzhou, 510060 People’s Republic of China; 3 Department of Cardiovascular Surgery, Sun Yat-sen Memorial Hospital, Sun Yat-sen University, Guangzhou, 510060 People’s Republic of China; 4 Department of Radiation Oncology, The First Affiliated Hospital of Sun Yat-sen University, Guangzhou, 510060 People’s Republic of China; 5 Department of Thoracic Oncology, Sun Yat-Sen University Cancer Center, Guangzhou, 510060 People’s Republic of China

**Keywords:** Rectal cancer, Stage IV, Neoadjuvant chemotherapy, Radiotherapy, Local treatment

## Abstract

**Background:**

The optimal treatment of stage IV rectal cancer remains controversial. The purpose of this study was to assess the treatment outcomes and toxicity of neoadjuvant chemotherapy and radiotherapy followed by local treatment of all tumor sites and subsequent adjuvant chemotherapy in stage IV rectal cancer patients with potentially resectable metastases.

**Methods:**

Adult patients diagnosed with locally advanced rectal adenocarcinoma with potentially resectable metastases, who received neoadjuvant chemotherapy and radiotherapy from July 2013 and September 2019 at Sun Yat-sen University cancer center, were included. Completion of the whole treatment schedule, pathological response, treatment-related toxicity and survival were evaluated.

**Results:**

A total of 228 patients were analyzed with a median follow-up of 33 (range 3.3 to 93.4) months. Eventually, 112 (49.1%) patients finished the whole treatment schedule, of which complete response of all tumor sites and pathological downstaging of the rectal tumor were observed in three (2.7%) and 90 (80.4%) patients. The three-year overall survival (OS) and progression-free survival (PFS) of all patients were 56.6% (50.2 to 63.9%) and 38.6% (95% CI 32.5 to 45.8%), respectively. For patients who finished the treatment schedule, 3-year OS (74.4% vs 39.2%, *P* < 0.001) and 3-year PFS (45.5% vs 30.5%, *P* = 0.004) were significantly improved compared those who did not finish the treatment. Grade 3–4 chem-radiotherapy treatment toxicities were observed in 51 (22.4%) of all patients and surgical complications occurred in 22 (9.6%) of 142 patients who underwent surgery, respectively.

**Conclusions:**

Neoadjuvant chemotherapy and radiotherapy followed by resection/ablation and subsequent adjuvant chemotherapy offered chances of long-term survival with tolerable toxicities for selected patients with potentially resectable stage IV rectal cancer, and could be considered as an option in clinical practice.

**Supplementary Information:**

The online version contains supplementary material available at 10.1186/s12885-021-09089-5.

## Background

Treatment of rectal cancer remains challenging with 15–25% of patients presenting synchronous metastases at diagnosis [1–3]. Approximately 80–90% of these metastases were initially unresectable with reported resection rates of merely 5–15% [4–6]. For patients with unresectable metastases, prognosis is poor.

In recent years, with the development of effective chemotherapeutic agents, the survival rate of stage IV rectal cancer significantly improved. In portion of these patients, effective conversion systemic chemotherapy could turn initially unresectable metastases into resectable [7]. Meanwhile, local treatment modalities were also developed. Improved surgical techniques, and the widely used of radiofrequency ablation (RFA) and stereotactic body radiotherapy (SBRT) offer a curative chance and do bring survival benefits in patients with stage IV rectal cancer [8, 9]. Aggressive multimodality therapy for patients with stage IV rectal cancer were to achieve the goal of no evidence of disease (NED) [10, 11]. However, for the locally advanced primary tumor and synchronous metastases, resection of all tumor sites is still challenging. Therefore, converting potentially resectable tumors into a resectable or ablationable disease is essential for these patients.

According to the National Comprehensive Cancer Network (NCCN) guidelines, neoadjuvant chemotherapy and radiotherapy followed by resection is the standard of care for stage II-III rectal cancer patients [12]. For patients with stage IV rectal cancer, pelvic radiotherapy is often used only as palliative care to relieve local symptoms [13, 14]. Systemic chemotherapy remains the cornerstone for stage IV rectal cancer [15]. For the majority of patients with primary lesions at the T3 or T4 stage, researchers are interested in whether adding neoadjuvant chemotherapy and radiotherapy could improve the resection rate, improve local control, and eventually lead to better survival [16]. In the Dutch phase II clinical trial [17] patients with stage IV rectal cancer received short-course radiotherapy (SCRT) followed by systemic chemotherapy and subsequent radical treatment. The results of the study showed that 72% of patients achieved R0 resection of both the primary tumor and metastases, and the 2-year overall survival (OS) was 80%. A respective study [18] with a similar design showed that 79.4% of patients achieved local symptom control and 78% had a chance at liver resection and/or RFA with a median OS of 51.5 months. These indicated that neoadjuvant radiotherapy might bring about some survival benefits in addition to chemotherapy for stage IV rectal cancer patients.

In this study, we assessed the treatment outcomes and toxicity of this multimodal treatment schedule, which consisted of neoadjuvant chemotherapy and radiotherapy, local treatment and adjuvant chemotherapy in patients with potentially resectable stage IV rectal cancer.

## Methods

### Patient population

We retrospectively reviewed the data of consecutive patients diagnosed with stage IV rectal cancer who received treatment at Sun Yat-sen University Cancer Center between July 2013 and September 2019. Their treatment plan was a multimodality schedule, including neoadjuvant chemotherapy, pelvic radiotherapy, followed by local treatment for both the primary tumor and metastases and subsequent adjuvant chemotherapy. The eligibility criteria were: (1) at least 18 years old; (2) pathologically confirmed rectal adenocarcinoma; (3) primary lesions were T1–2 with positive regional lymph nodes or T3–4 with both positive and negative lymph nodes; (4) synchronous potentially resectable metastases (including liver, lung and/or distant lymph nodes) located in no more than two organs; and (5) a Karnofsky Performance Status of at least 70. Patients were excluded if they: (1) underwent primary tumor resection or metastasectomy before neoadjuvant chemotherapy and radiotherapy; (2) had a prior history of other malignancies within five years; (3) severe diseases including heat, brain, lung, liver or kidney dysfunction; or (4) metastasis to the peritoneum.

In clinical practice, an assessment of metastases resectability was conducted by a multidisciplinary team (MDT) consisting of radiologists, surgeons, and radiation oncologists. The criteria for potentially resectable liver metastases in the study were defined as: satisfactory margins after resection or the residual liver volume preserved is >30% after conversion therapy. Criteria for potentially resectable pulmonary metastases were defined as: adjacent to vital structures, such as the great vessels, heart, esophagus or centrum, which can be treated with conversion therapy to obtain R0 resection. In addition, patients with distant lymph node metastases vary greatly, and there is no uniformity in the criteria for resectability. It is related to the experience and surgical skills of the supervising surgeons.

This research was approved by the Research Ethics Committees of Sun Yat-sen University cancer center (B2021–089-01).

### Pretreatment assessment

Pretreatment assessment consisted of a complete physical examination, carcinoembryonic antigen (CEA) and carbohydrate antigen 19–9 (CA-199) level tests, colonoscopy with pathological examination, enhanced magnetic resonance imaging (MRI) or enhanced computerized tomography (CT) of the pelvis (CT was performed only in patients with a contraindication to MRI), and enhanced CT of the chest and abdomen. MRI of the liver was optional when liver metastasis was suspected and was performed at the discretion of the attending physician. Risk factors considered were patient age (≥60 or < 60 years), gender (male or female), cT category (IV or II-III stage), cN category (II or 0-I stage), CEA (> 5 or ≤ 5 ng/ml), CA199 (> 35 or ≤ 35 U/ml), metastatic organs (multiple or single) and number of liver metastases (> 5 vs ≤ 5). The 8th edition of the American Joint Committee on Cancer (AJCC) was used to stage patients.

### Neoadjuvant treatment and reassessment

The treatment schedule for all patients was determined via an MDT. Chemotherapy started immediately after diagnosis, with a regimen of CAPOX (capecitabine and oxaliplatin); FOLFOX (fluorouracil, folinic acid and oxaliplatin); or capecitabine monotherapy according to the performance status of each patient and at the direction of the treating physicians. Neoadjuvant radiotherapy included long-course radiotherapy (LCRT) or SCRT given to the rectal tumor, mesorectum and metastatic lymph nodes. Intensity-modulated technology was applied in this study. LCRT consisted of 50Gy delivered in 25 fractions and was started concurrently with the 2nd cycle of chemotherapy. Five weeks after LCRT, patients were reassessed by colonoscopy, an enhanced CT scan of chest and abdomen, an enhanced MRI of the pelvis and a CEA level test. SCRT consisted 25Gy delivered in 5 fractions and was given after finishing 4–6 cycles of chemotherapy and no concurrent chemotherapy was given. Reassessment was performed before the decision to receive SCRT (Supplementary Fig. 1).

### Surgery and postoperative adjuvant chemotherapy

After reassessment, the ability to resect the primary tumor and metastases was discussed by the MDT. According to the examination results, the MDT determined the next procedure: palliative treatment, staggered or concurrent local treatment of rectal cancer and/or distant metastases, or continued systemic chemotherapy.

Rectum resection was scheduled within one week after SCRT or 6–8 weeks after LCRT for total mesorectal excision (TME). For metastatic lesions, metastasectomy was the preferred technique. Other treatment modalities included RFA and SBRT, which were optional. After local treatment of all tumor sites, adjuvant chemotherapy was offered with the same regimen as in the neoadjuvant settings. The planed duration of perioperative chemotherapy was to reach a total of 6 months (8 cycles for the 3-weekly regimen or 12 cycles for the 2-weekly regimen). The histopathological assessment of resection specimens was conducted by pathologists, and the post-neoadjuvant pathological response was evaluated using Mandard’s classification.

### Outcomes and follow-up

The primary outcome was OS. Secondary outcomes were progression-free survival (PFS), completion of the whole treatment schedule, pathological response, treatment toxicity, and surgical complications The tumor response assessment was conducted using the Response Evaluation Criteria in Solid Tumors (RECIST) version 1.0. Treatment toxicity was recorded using the Common Terminology Criteria for Adverse Events (CTCAE) version 4.0. Postoperative complications were evaluated in accordance with the Clavien-Dindo classification.

All patients were followed up every three months during the first two years, semiannually over the next three years, then annually for the following years. The last date of follow up was in March 2021. OS was evaluated from the date of the first treatment to death from any cause or censored at the last follow-up. PFS was calculated from the date of the first treatment until the diagnosis of the first documented local or distant progression, or death related to rectal cancer, whichever came first. Completion of the treatment schedule was defined as finishing neoadjuvant chemotherapy and radiotherapy, subsequent local treatment for all tumor sites and at least 2 cycles of adjuvant chemotherapy.

### Statistical analysis

The statistical analysis was carried out with SPSS version 26.0 (IBM, Armonk, NY, USA) and R version 4.0.4 (http://www.Rproject.org). Statistical data were reported as medians with ranges, and the categorical data were reported in proportions. Survival curves were displayed by Kaplan-Meier analysis, and the survival rates compared by using the log-rank test. A Cox proportional hazards regression was used to examine the independent prognostic factors by calculating the hazard ratios (HR) and 95% confidence intervals (CI) in univariate and multivariate analyses. A *p* value of < 0.05 was considered significant.

## Results

### Patient characteristics

**Fig. 1:**
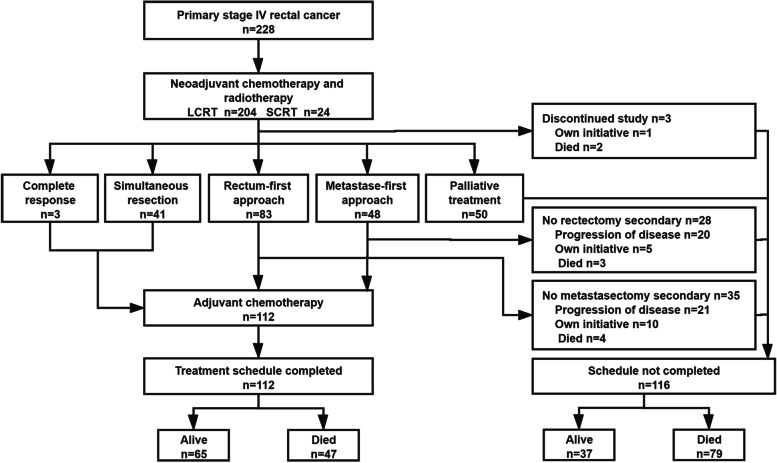
Flowchart for all patients in the study. Abbreviations: LCRT, long-course radiotherapy; SCRT, short-course radiotherapy

From July 2013 to September 2019, a total of 228 patients with primary stage IV rectal cancer who met the criteria were given the multimodality treatment schedule (Fig. 1). Patients and treatment characteristics are presented in Tables 1 and 2 and Supplementary Table 1. The median follow up was 33 (range 3.3 to 93.4) months.

**Table 1:** Patient demographics and disease characteristics (*n* = 228)

Characteristics	No. of patients (%) ^b^
Age at start of treatment ^a^ (years)	56 (25–81)
Sex ratio (M: F)	160: 68
KPS	
70–80	26 (11.4)
90–100	202 (88.6)
Clinical tumor category	
T2 N1–2	2 (0.9)
T3 N0	2 (0.9)
T3 N1–2	95 (41.7)
T4 N1–2	129 (56.6)
Metastatic site	
Liver	116 (50.9)
Lung	64 (28.1)
Liver and lung	20 (8.8)
Distant lymph nodes	28 (12.3)
No. of liver metastases	*n* = 116
1–3	78 (67.2)
4–5	9 (7.8)
≥ 6	29 (25)
Location of liver metastases	*n* = 136
Unilobar	52 (38.2)
Multilobar	84 (61.8)
Diameter of largest liver metastasis ^a^ (cm)	2.0 (0.6–12)
Length of rectal cancer ^a^ (cm)	5.5 (1.5–16)
Location of primary rectal cancer	
Low (0-5 cm)	61 (26.8)
Middle (5-10 cm)	134 (58.8)
High (10-15 cm)	33 (14.5)
Differentiation	
Well	9 (3.9)
Moderate	177 (77.6)
Poor	31 (13.6)
Unknown	11 (4.8)
CEA at diagnosis ^a^ (ng/ml)	14.6 (0.7–2677)
CA199 at diagnosis ^a^ (U/ml)	31.4 (0.6–20,000)

^a^ Values are median (range). ^b^With percentages in parentheses unless indicated otherwise

*Abbreviations*: *KPS* Karnofsky Performance Status, *CEA* carcinoembryonic antigen, *CA199* carbohydrate antigen 19–9

**Table 2:** Treatment details (n = 228)

Characteristics	No. of patients (%)
Radiotherapy	
25 × 2Gy	204 (89.5)
5 × 5Gy	24 (10.5)
First-line chemotherapy	
CAPOX	180 (78.9)
CAPOX-B	9 (3.9)
FOLFOX	10 (4.4)
FOLFOX-B	7 (3.1)
CAP	15 (6.6)
CAP-B	2 (0.9)
Other	5 (2.2)
Time of chemotherapy (months)	
≤ 3	23 (10.1)
3–6	131 (57.5)
> 6	74 (32.5)
Rectal resection	*n* = 142
Low anterior resection	116 (81.7)
Abdominoperineal resection	20 (14.1)
Hartmann procedure	6 (4.2)
Diverting stoma	*n* = 73
Simultaneous surgery	50 (68.5)
During chemotherapy	15 (20.5)
Before treatment	8 (11.0)
pTRG (Mandard) ^a^	*n* = 129
Complete regression (TRG 1)	25 (19.4)
Good regression (TRG 2)	49 (38.0)
Moderate regression (TRG 3)	41 (31.8)
Slight regression (TRG 4)	14 (10.9)
Liver treatment	*n* = 85
Liver resection	30 (35.3)
RFA	33 (38.8)
Resection + RFA	18 (21.2)
Radiotherapy	4 (4.7)
Treatment of extrahepatic metastases	
Lung	
RFA	22 (9.6)
Metastasectomy	7 (3.1)
Radiotherapy	2 (0.9)
Lymph node resection	6 (2.6)
Rectal radiotherapy expanded for lymph node	28 (12.3)

^a^ pathological tumor regression grade of 5-tier Mandard adjuvant

*Abbreviations*: *B* bevacizumab, *RFA* radiofrequency ablation, *CAP* capecitabine, *OX* oxaliplatin; FOLFOX, 5-fluorouracil, folinic acid and oxaliplatin

The most common site for metastases was the liver (116 patients, 50.9%), followed by the lungs (64 patients, 28.1%), both liver and lungs (20 patients, 8.9%) as well as distant lymph nodes including the paraaortic lymph nodes and the left supraclavicular lymph nodes (28 patients, 12.3%).

### Completion of treatment schedule

All patients received neoadjuvant chemotherapy and pelvic intensity-modulated radiation therapy (IMRT). LCRT and SCRT were given to 204 patients (89.5%) and 24 patients (10.5%), respectively. After neoadjuvant treatment, 100 patients (41.0%) received a bowel-first approach, 20 patients (8.2%) received a metastases first approach and simultaneous resection was performed in 37 patients (15.2%). Three patients (1.2%) had a clinical complete response (cCR) to both the primary rectal tumor and distant metastases. The number of patients who finished the whole treatment schedule was 112 (49.1%) (Fig. 1).

Of the 116 patients who were not able to complete the treatment schedule, 50 patients (21.9%) received palliative treatment, 33 patients (14.5%) received bowel surgery only, 25 patients (11.0%) received local metastases treatment only, one patient (0.4%) rejected radical treatment and 7 patients (3.1%) died of metastases progression during treatment.

### Overall and progression-free survival

Seventy patients (30.7%) developed progressive diseases during treatment and 79 patients (34.6%) had disease progression after treatment to the last follow up or death for the whole cohort. Liver and lung were the most common sites of the progression. Only 7 patients (3.1%) had local progression, of them, three occurred during treatment and four occurred after treatment (Table 3). The median OS for all patients was 41.7 (range 3.3 to 93.4) months and the 1–2-and 3-year OS rates were 91.2% (95% CI 87.6 to 95%), 74.8% (69.4 to 80.7%) and 56.6% (50.2 to 63.9%), respectively. Median PFS for all patients was 20.5 (range 0.7 to 93.4) months and the 1–2-and 3-year PFS rates were 67.4% (95% CI, 61.5 to 73.8%), 43.7% (95% CI, 37.6 to 50.7%) and 38.6% (95% CI, 32.5 to 45.8%), respectively.

**Table 3:** Location of progression of disease during and after treatment

Location	During treatment	After treatment
Liver	24	23
Lung	18	27
Rectum	3	4
Liver and lung	11	6
Liver, lung and lymph nodes	3	5
Peritoneum	5	4
Bone	4	3
Other	2	7

**Fig. 2:**
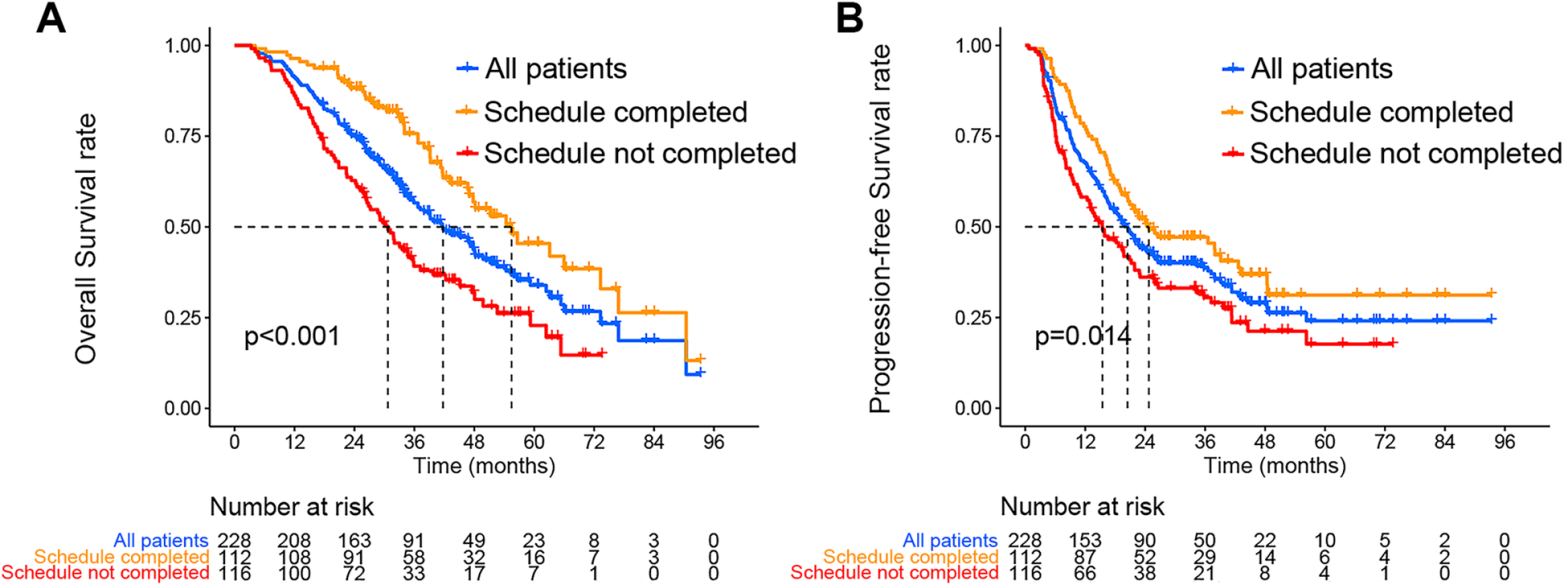
Kaplan–Meier analysis of overall survival (**A**) and progression-free survival (**B**) for the whole cohort, patients who completed the treatment schedule, and those who did not

In the subgroup analysis, we found that the median OS of the 112 patients who completed the whole treatment schedule was 55.5 months, compared with 30.7 months for patients who did not complete treatment (*P* < 0.001). The 1–2 and 3-year OS rates for patients who completed the whole treatment schedule were 96.4% (95% CI, 61.5 to 73.8%), 88.2% (95% CI, 82.4 to 94.5%) and 74.4% (95% CI, 66.1 to 83.8%), respectively. The median PFS of the 112 patients was 24.7 (range 0.8 to 93.4) months, and the 1–2-and 3-year PFS rates were 76.8% (95% CI, 69.4 to 85.0%), 51.4% (95% CI, 42.8 to 61.6%) and 45.5% (95% CI, 36.8 to 56.2%), respectively. For patients who finished the treatment schedule, 3-year OS (74.4% vs 39.2%, P < 0.001) and 3-year PFS (45.5% vs 30.5%, *P* = 0.004) were significantly improved compared those who did not finish the treatment (Fig. 2).

In the multivariable analysis, the number of liver metastases (HR 1.18, 95% CI 0.300 to 2.866, *P* = 0.028) was the only factor associated with decreased PFS among the whole cohort (Table 4). And all risk factors were not statistically significant on multivariable analysis for OS (Supplementary Table 2).

**Table 4:** Univariable and multivariable analysis for progression-free survival

	Univariable analysis		Multivariable analysis	
Factor	Hazard ratio	P	Hazard ratio	P
Age (> 60 vs ≤ 60 ^a^)	1.15 (0.72, 1.84)	0.553		
Sex (M vs F ^a^)	1.04 (0.65, 1.67)	0.865		
cT category (IV vs II-III ^a^)	1.62 (0.62, 2.58)	0.045	0.98 (0.62, 1.54)	0.916
cN category (II vs 0-I ^a^)	1.07 (0.94, 1.73)	0.791		
CEA (> 5 or ≤ 5 ^a,b^)	1.17 (0.71, 1.92)	0.531		
CA199 (> 35 or ≤ 35 ^a,c^)	1.02 (0.65, 1.61)	0.929		
Metastatic organs (Multiple vs single ^a^)	1.06 (0.42, 2.64)	0.089		
No. of liver metastases (> 5 vs ≤ 5 ^a^)	1.25 (0.96, 2.84)	0.008	1.18 (0.30, 2.89)	0.028

^a^ The control group of multivariate Cox analysis. ^b^ The normal values for CEA range 0–5 ng/ml. ^c^ The normal values for CA199 range 0–35 U/ml

*Abbreviations*: *M* male, *F* female, *cT category* clinical T category, *cN category* clinical N category, *CEA* carcinoembryonic antigen, *CA199* carbohydrate antigen 19–9

### Evaluation of radiological and pathological responses

The cCR of all the tumor sites were observed in three out of 228 patients (1.2%) and they were given a watch and wait strategy after a total of six months of chemotherapy. One of these patients had liver recurrence five months after finishing treatment and was given palliative treatment. Other patients did not experience recurrence during a median follow-up of 32 months. Clinical partial response occurred in 130 patients (57.0%) after neoadjuvant chemotherapy and radiotherapy. Primary rectal surgery was conducted in 142 patients (62.3%) and R0 resection was achieved in 137 patients (96.5%). Of these patients, 25 (17.6%) presented a pathological complete response (pCR) and 74 (52.1%) showed a good pathological tumor regression grade (TRG1 and TRG2). They were given postoperative chemotherapy. Pathological downstaging of the rectal tumor was observed in 90 patients (80.4%). Surgical resection of liver and lung metastases were conducted in 48 (21.1%) and seven (3.1%) patients, with a pCR of the liver and lung were respectively reported in six (12.5%) and one (14.3%) patient among them. The imaging characteristics of stage IV rectal cancer patients with liver or lung metastases after neoadjuvant chemotherapy and radiotherapy are presented in Supplementary Fig. 2.

### Toxicities and complications

Grade 3–4 toxicities were observed in 50 patients (21.9%) during neoadjuvant treatment, with hematological toxicity reported mostly frequently in 27 patients (11.8%) and gastrointestinal toxicity reported in 13 patients (5.7%). For hematological toxicity, 14 and 13 patients had thrombocytopenia and neutropenia, respectively. Patients were given symptomatic management and all completed neoadjuvant treatment. Surgical complications were observed in 22 patients (9.6%) (Table 5). The most common complications were intestinal obstruction observed in 14 patients (6.1%), with only six of them requiring surgical intervention. Five patients (2.2%) had anastomotic leakage and four of them were treated with enterostomy. No patients died as a result of surgical side effects.

**Table 5:** Neoadjuvant treatment toxicity and local treatment complications

Events	Grade 3	Grade 4	Total no. of patients
Chemoradiotherapy-related toxicity			
Hematological	23	4	27
Gastrointestinal	10	3	13
Neurological	5	0	5
Dermatological	3	2	5
Surgical complications			
Intestinal obstruction	8	6	14
Anastomotic leakage	5	0	5
Anastomotic stenosis	2	0	2
Abscess	1	0	1

## Discussion

Currently, the optimal treatment of stage IV rectal cancer remains unclear [19]. In the context of a locally advanced primary tumor with synchronous metastasis, an effective treatment schedule should combine locoregional control with an adequate dose of systemic chemotherapy for all tumor sites [20]. In this present study, neoadjuvant chemotherapy and radiotherapy followed by resection/ablation and subsequent adjuvant chemotherapy achieved long-term survival with a relatively low recurrence rate and tolerable toxicities in rectal cancer patients with potentially resectable synchronous metastases, demonstrating the feasibility of this treatment schedule.

Neoadjuvant chemotherapy and radiotherapy provide survival benefit over chemotherapy alone in down-staging of the primary lesion and in converting potentially resectable metastases into a resectable or ablatable disease. In a national database analysis [21] conducted with 4051 patients with metastatic rectal adenocarcinoma, the median OS was 46.3 months versus 35.3 months in favor of additional radiotherapy. In a phase II clinical trial [22], 32 patients with synchronous unresectable metastases of rectal cancer underwent radical surgery of the primary tumor and 85.7% of them showed a pathological down-staging of the primary tumor after neoadjuvant chemotherapy and radiotherapy. In addition, 35.7% of these patients had a pathological complete response. However, in a small sample sized piece of research [23], Milito P et al. reported a 3-year local regional recurrence-free survival (LRRFS) rate of 80.3% for neoadjuvant chemotherapy and radiotherapy vs. 90.4% for upfront surgery patients (*P* = 0.35). This is inconsistent with our results. Nevertheless, this inconsistency may be due to differences in patient selection. In Milito P’s study, only patients with a resectable primary tumor were included. In contrast, in this present study, most patients had a locally advanced primary tumor and potentially resectable metastases at baseline and the opportunity of radical resection for all tumor sites depended on the response to neoadjuvant chemotherapy and radiotherapy. Eventually, 134 patients (58.9%) received local treatment for metastases and 142 patients (62.3%) received radical rectal surgery, of which more than half of them (57.4%) were pathologically confirmed as having good regression of the rectal tumor. In our study, pelvic radiation significantly reduced the risk of rectal bleeding and obstruction during treatment compared with previous studies [24, 25] and contributed to the relatively low recurrence rate after the whole treatment schedule.

Local treatment of metastases and resection of primary tumors could diminish tumor burden and reach a clinically NED for patients. As is well known, surgical resection, RFA and SBRT are common local treatments for metastases, and earlier studies [26, 27] have shown that there are no differences in survival time among them. Currently, there is a trend to adopt the watch and wait strategy for complete responders of locally advanced rectal cancer after neoadjuvant chemoradiotherapy. Our previous study [28] showed that compared to standard TME, the watch and wait strategy got similar survival outcomes and a superior sphincter preservation for stage II/III rectal cancer patients. However, all the available data discussing this topic currently applied only to patients without distant metastasis. As the adding of radiotherapy and more cycles of chemotherapy, or even total neoadjuvant chemotherapy in stage IV diseases, there are also chances of complete response in primary tumor. In this study, complete response was observed in 19.4% of patients who underwent surgical resection of the primary tumor and the 3-year OS was up to 91.4%. For these patients, the watch and wait strategy might be an option, especially for distal rectal cancers. Clinical trials discussing the watch-and-wait strategy for this subgroup of patients are warranted to verify this hyposis.

Recurrence of distant metastasis remains the main treatment problem for these patients. The high rate of early recurrence is consistent with other research [29, 30], with nearly half of patients developing disease recurrence within two years of starting treatment. In view of the high recurrence and poor prognosis of stage IV rectal cancer, most patients were offered a total of six months chemotherapy to eliminate small and potentially occult metastases early in this present study. However, there were still 34.6% of patients who had disease progression after treatment. Immunotherapy, targeted therapies and triplet chemotherapy which might facilitate the removal of potential micrometastasis and the strategy of total neoadjuvant therapy could be considered, to improve the treatment efficacy [31, 32].

This treatment schedule was relatively safe and tolerable, even though long-term radiotherapy, multi-course chemotherapy and various local treatments were given to the majority of patients. Bone marrow suppression and gastrointestinal reactions including nausea, diarrhea and ileus were the most common side effects during radiotherapy and chemotherapy, which is consistent with the results from previous research [33]. In this study, all patients finished the full dose of radiotherapy without intolerable toxic effects. Similar with other studies [34, 35], anastomotic leakage was the most frequently reported surgical complication apart from intestinal obstruction, but most were manageable with a diverting colostomy. No lethal adverse events occurred throughout this study’s entire treatment schedule.

To the best of our knowledge, this study comprised of one of the largest stage IV rectal cancer patient cohorts who underwent neoadjuvant chemotherapy and radiotherapy followed by radical surgery and local treatment, with clinical values for subsequent research. However, we acknowledge there are some limitations of this study. First, the assessment of potentially resectable metastases remains unclear and was influenced by subjective options. In this study, the treatment of all patients with potentially resectable metastases was discussed by the MDT group, and this, to some extent made the assessment more objective. Second, as a retrospective investigation conducted in a single center, this study had inherent selection bias. Large prospective randomized controlled clinical trials are needed to verify these results to provide the optimal treatment schedule for these patients.

## Conclusions

Neoadjuvant chemotherapy and radiotherapy followed by surgery/ablation and adjuvant chemotherapy offered chances of long-term survival with tolerable toxicities in potentially resectable stage IV rectal cancer patients, and could be considered as an option in clinical practice.

## Supplementary Information

**Additional file 1: Supplementary Figure 1**. The most common treatment modal of neoadjuvant chemotherapy and radiotherapy.

**Additional file 2: Supplementary Figure 2**. Representative images before and after neoadjuvant therapy in stage IV rectal cancer patients with synchronous metastases (red arrows). A. MRI imaging showing the disappearance of synchronous liver metastases after neoadjuvant therapy. B. CT imaging showing the disappearance of synchronous lung metastases after neoadjuvant therapy.

**Additional file 3: Supplementary Table 1**. Patient demographics and disease characteristics (*n* = 228).

**Additional file 4: Supplementary Table 2**. Univariable and multivariable analysis for overall survival.

## Data Availability

All data are available via the corresponding author.

## Abbreviations

OS: Overall survival
PFS: Progression-free survival
RFA: Radiofrequency ablation
SBRT: Stereotactic body radiotherapy
NCCN: National Comprehensive Cancer Network
SCRT: Short-course radiotherapy
CEA: Carcinoembryonic antigen
CA-199: Carbohydrate antigen 19–9
AJCC: American Joint Committee on Cancer
MDT: Multidisciplinary team
CAPOX: Capecitabine and oxaliplatin
FOLFOX: Fluorouracil, folinic acid and oxaliplatin
LCRT: Long-course radiotherapy
TME: Total mesorectal excision
RECIST: Response Evaluation Criteria in Solid Tumors
CTCAE: Common Terminology Criteria for Adverse Events
HR: Hazard ratios
CI: Confidence intervals
cCR: Clinical complete response
pCR: Pathological complete response
TRG: Tumor regression grade
NED: No evidence of disease
LRRFS: Local regional recurrence-free survival
