# Supplementary figures and images for: Neoadjuvant chemotherapy and radiotherapy followed by resection/ablation in stage IV rectal cancer patients with potentially resectable metastases
Source: BMC Cancer. 2021 Dec 14;21:1333. doi: 10.1186/s12885-021-09089-5 (PMC8672531; doi:10.1186/s12885-021-09089-5)

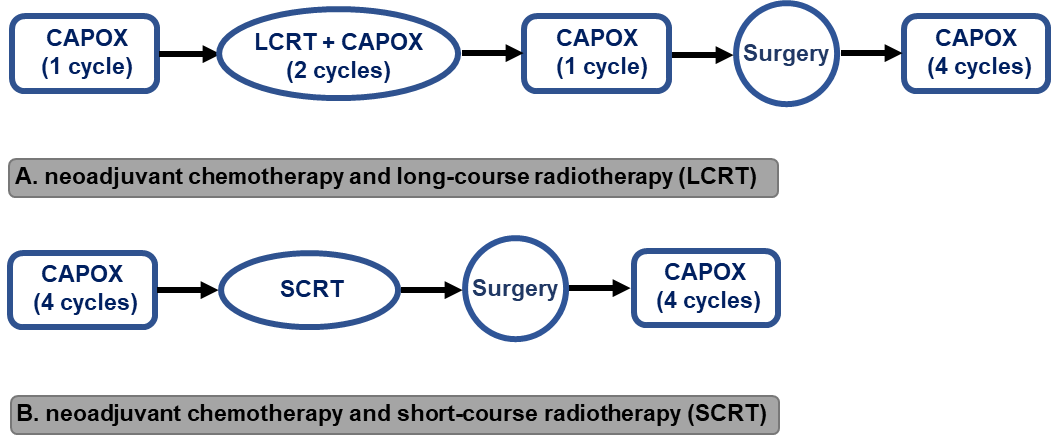

Supplement: Supplementary file 1 — Additional file 1: Supplementary Figure 1. The most common treatment modal of neoadjuvant chemotherapy and radiotherapy. [file 12885_2021_9089_MOESM1_ESM.tif]

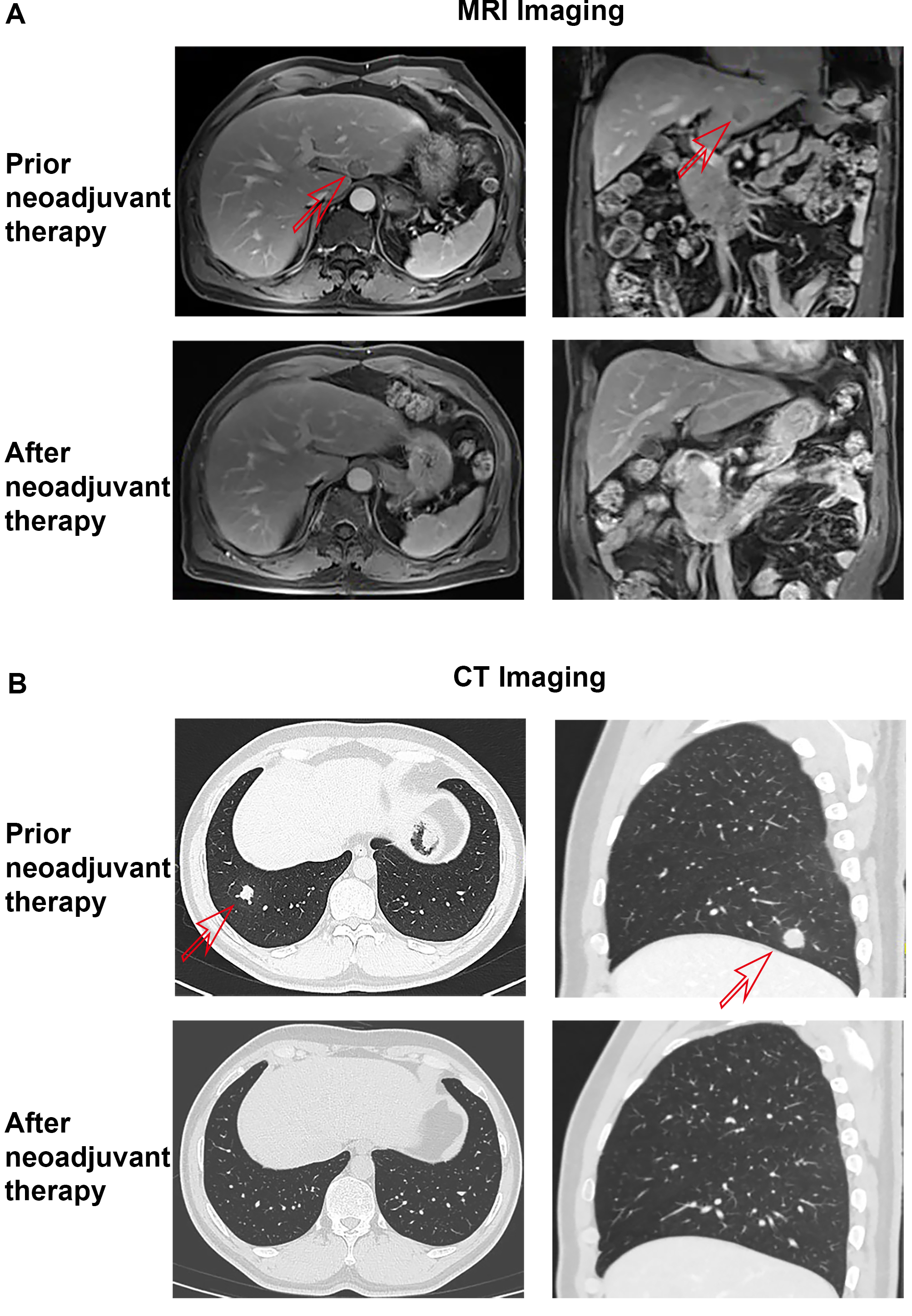

Supplement: Supplementary file 2 — Additional file 2: Supplementary Figure 2. Representative images before and after neoadjuvant therapy in stage IV rectal cancer patients with synchronous metastases (red arrows). A. MRI imaging showing the disappearance of synchronous liver metastases after neoadjuvant therapy. B. CT imaging showing the disappearance of synchronous lung metastases after neoadjuvant therapy. [file 12885_2021_9089_MOESM2_ESM.tif]
