# Supplementary material for: Neoadjuvant chemotherapy and radiotherapy followed by resection/ablation in stage IV rectal cancer patients with potentially resectable metastases
Source: BMC Cancer. 2021 Dec 14;21:1333. doi: 10.1186/s12885-021-09089-5 (PMC8672531; doi:10.1186/s12885-021-09089-5)
Supplement: Supplementary file 3 — Additional file 3: Supplementary Table 1. Patient demographics and disease characteristics (n = 228). [file 12885_2021_9089_MOESM3_ESM.docx]

| **Characteristics** | **No. of patients (%) ^b^** | | | **P** | |
| --- | --- | --- | --- | --- | --- |
|  | **All**  **(n=228)** | **Completed (n=112)** | **Not completed**  **(n=116)** | |  |
| Age at start of treatment ^a^ (years) | 56 (25-81) | 56 (25-80) | 56 (25-81) | | 0.608 |
| Sex ratio (M: F) | 160: 68 | 78: 34 | 82: 34 | | 0.978 |
| KPS |  |  |  | | 0.382 |
| <90 | 26 (11.4) | 8 (7.1) | 13 (11.4) | |  |
| ≥90 | 202 (88.6) | 104 (92.9) | 101 (88.6) | |  |
| Clinical tumor category |  |  |  | | 0.520 |
| T2 N1-2 | 2 (0.9) | 1 (0.9) | 1 (0.9) | |  |
| T3 N0 | 2 (0.9) | 0 (0.0) | 2 (1.7) | |  |
| T3 N1-2 | 95 (41.7) | 45 (40.2) | 50 (43.1) | |  |
| T4 N1-2 | 129 (56.6) | 66 (58.9) | 63 (54.3) | |  |
| Metastatic site |  |  |  | | <0.001 |
| Liver | 116 (50.9) | 70 (62.5) | 46 (39.7) | |  |
| Lung | 64 (28.1) | 19 (17.0) | 45 (38.8) | |  |
| Liver and lung | 20 (8.8) | 6 (5.4) | 14 (12.1) | |  |
| Distant lymph nodes | 28 (12.3) | 17 (15.2) | 11 (9.5) | |  |
| No. of liver metastases | n=116 |  |  | | 0.001 |
| 1-3 | 78 (67.2) | 56 (80.0) | 22 (47.8) | |  |
| 4-5 | 9 (7.8) | 5 (7.1) | 4 (8.7) | |  |
| ≥ 6 | 29 (25) | 9 (12.9) | 20 (43.5) | |  |
| Location of liver metastases | n=136 |  |  | | 0.105 |
| Unilobar | 52 (38.2) | 36 (46.2) | 19 (31.1) | |  |
| Multilobar | 84 (61.8) | 42 (53.8) | 42 (68.9) | |  |
| Diameter of largest liver metastasis ^a^ (cm) | 2.0 (0.6-12) | 1.8 (0.6-7.5) | 2.4 (0.6-12) | | 0.005 |
| Length of rectal cancer ^a^ (cm) | 5.5 (1.5-16) | 5.6 (2.5-12) | 5.2 (1.5-16) | | 0.934 |
| Location of primary rectal cancer |  |  |  | | 0.358 |
| Low (<5cm) | 54 (23.7) | 22 (19.6) | 32 (27.6) | |  |
| Middle (5-10cm) | 132 (57.9) | 69 (61.6) | 63 (54.3) | |  |
| High (>10cm) | 42 (18.4) | 21 (18.8) | 21 (18.1) | |  |
| Differentiation |  |  |  | | 0.503 |
| Well | 9 (3.9) | 3 (2.7) | 6 (5.2) | |  |
| Moderate | 177 (77.6) | 93 (83.0) | 84 (72.4) | |  |
| Poor | 31 (13.6) | 15 (13.4) | 16 (13.8) | |  |
| Unknown | 11 (4.8) | 1 (0.9) | 10 (8.6) | |  |
| CEA at diagnosis ^a^ (ng/ml) | 14.6 (0.7-2677) | 14.9 (0.7-791) | 14.5 (1.29-2677) | | 0.037 |
| CA199 at diagnosis ^a^ (U/ml) | 31.4 (0.6-20000) | 26.35 (1.6-12647) | 36.1 (0.6-20000) | | 0.329 |

^a^ Values are median (range). ^b^With percentages in parentheses unless indicated otherwise. Abbreviations: KPS, Karnofsky Performance Status; CEA, carcinoembryonic antigen; CA199, carbohydrate antigen 19-9.
