# Supplementary material for: Neoadjuvant chemotherapy and radiotherapy followed by resection/ablation in stage IV rectal cancer patients with potentially resectable metastases
Source: BMC Cancer. 2021 Dec 14;21:1333. doi: 10.1186/s12885-021-09089-5 (PMC8672531; doi:10.1186/s12885-021-09089-5)
Supplement: Supplementary file 4 — Additional file 4: Supplementary Table 2. Univariable and multivariable analysis for overall survival. [file 12885_2021_9089_MOESM4_ESM.docx]

|  | **Univariable analysis** | |  | **Multivariable analysis** | |
| --- | --- | --- | --- | --- | --- |
| **Factor** | **Hazard ratio** | **P** |  | **Hazard ratio** | **P** |
| Age (≥ 60 vs < 60 ^a^) | 1.01 (0.99, 1.02) | 0.424 |  |  |  |
| Sex (M vs F ^a^) | 1.12 (0.75, 1.67) | 0.591 |  |  |  |
| cT category (IV vs II-III ^a^) | 1.24 (0.86, 1.78) | 0.261 |  |  |  |
| cN category (II vs 0-I ^a^) | 1.04 (0.72, 1.51) | 0.826 |  |  |  |
| CEA (> 5 or ≤ 5 ^a,b^) | 1.59 (0.99, 2.54) | 0.056 |  |  |  |
| CA199 (> 35 or ≤ 35 ^a,c^) | 1.64 (1.14, 2.36) | 0.008 |  | 1.24 (0.69, 2.24) | 0.470 |
| Metastatic organs (multiple vs single ^a^) | 1.03 (0.57, 1.87) | 0.925 |  |  |  |
| No. of liver metastases (> 5 vs ≤ 5 ^a^) | 1.81 (1.07, 3.08) | 0.028 |  | 0.97 (0.54, 1.74) | 0.923 |

^a^ The control group of multivariate Cox analysis. ^b^ The normal values for CEA range 0-5 ng/ml. ^c^ The normal values for CA199 range 0-35 U/ml. Abbreviations: M，male; F, female; cT category, clinical T category; cN category, clinical N category; CEA, carcinoembryonic antigen; CA199, carbohydrate antigen 19-9.
